# Mental Health Stigma and Psychological Well-Being in Abu Dhabi, United Arab Emirates: A Population-Based Study

**DOI:** 10.1192/bjo.2026.11068

**Published:** 2026-06-30

**Authors:** Syed Fahad Javaid, Huda Saeed Obaid Alshemeili, Javaid Nauman, Luai Ahmed, Iffat Elbarazi

**Affiliations:** 1 Department of Psychiatry, College of Medicine and Health Sciences, United Arab Emirates University, Al Ain, UAE; 2 Abu Dhabi Public Health Centre, Abu Dhabi, UAE; 3 Institute of Public Health, College of Medicine and Health Sciences, United Arab Emirates University, Al Ain, UAE

## Abstract

**Aims:**

Mental health-related stigma is a recognised determinant of psychological well-being,yet population-level evidence from the United Arab Emirates (UAE) remains limited. This study aimed to assess levels of perceived mental health stigma among residents of Abu Dhabi, examine socio-demographic correlates of stigma, and explore the relationship between stigma and psychological well-being among Emirati and expatriate populations.

**Methods:**

A cross-sectional, population-based study was conducted among adult residents of the Abu Dhabi region. Participants completed a culturally adapted and validated Stigma-9 (STIG-9) questionnaire to assess perceived public stigma, and the World Health Organization Five Well-Being Index (WHO-5) to measure psychological well-being. Socio-demographic variables included age, gender, region of residence, nationality, education level, and household income. Descriptive statistics summarised stigma and well-being scores. Group comparisons examined demographic differences, and correlation analysis assessed the relationship between stigma and well-being. Statistical significance was set at p<0.05.

**Results:**

Overall perceived stigma levels were low (mean STIG-9 score 0.92 ± 0.89; median 0.89, IQR 0–1.67). Significant regional variation was observed, with participants residing in Al Ain reporting higher stigma scores than those in other regions. Females reported higher stigma than males (1.02 ± 0.89 vs 0.83 ± 0.88; p<0.001). Younger adults aged 18–29 years also demonstrated higher stigma scores (1.04 ± 0.93) compared with older age groups. Higher education level and higher household income were associated with increased perceived stigma, while no significant differences were observed by nationality.

Psychological well-being scores were generally high (mean WHO-5 score 74.21 ± 25.60; median 75, IQR 55–90). Males reported higher well-being than females (77.79 ± 24.90 vs 70.57 ± 25.79; p<0.001). Participants in the lowest household income group (<5,000 AED) reported the highest well-being scores (81.01 ± 24.99), while participants with postgraduateeducation reported lower well-being (60.77 ± 21.58). A strong inverse association was identified between perceived stigma and psychological well-being (r=−0.67, 95% CI −0.70 to −0.65; p<0.001).

**Conclusion:**

While the study participants generally showed low perceived mental health stigma, notable variation existed across demographics and regions. Higher stigma was seen among younger adults, females, and those with higher socioeconomic status, reflecting complex sociocultural influences. The inverse link between stigma and well-being emphasises the need for stigma-reduction strategies as part of mental health promotion. Culturally tailored interventions for high-risk groups could support psychological well-being across UAE communities.

